# Wearable Sensors for Healthcare: Fabrication to Application

**DOI:** 10.3390/s22145137

**Published:** 2022-07-08

**Authors:** Subhas Chandra Mukhopadhyay, Nagender Kumar Suryadevara, Anindya Nag

**Affiliations:** 1 School of Engineering, Macquarie University, Sydney, NSW 2109, Australia; subhas.mukhopadhyay@mq.edu.au; 2 School of Computer and Information Sciences, University of Hyderabad, Hyderabad 500046, India; nks@uohyd.ac.in; 3 Faculty of Electrical and Computer Engineering, Technische Universität Dresden, 01062 Dresden, Germany; 4 Centre for Tactile Internet with Human-in-the-Loop (CeTI), Technische Universität Dresden, 01069 Dresden, Germany

**Keywords:** wearable, sensors, healthcare, flexible, IoT

## Abstract

This paper presents a substantial review of the deployment of wearable sensors for healthcare applications. Wearable sensors hold a pivotal position in the microelectronics industry due to their role in monitoring physiological movements and signals. Sensors designed and developed using a wide range of fabrication techniques have been integrated with communication modules for transceiving signals. This paper highlights the entire chronology of wearable sensors in the biomedical sector, starting from their fabrication in a controlled environment to their integration with signal-conditioning circuits for application purposes. It also highlights sensing products that are currently available on the market for a comparative study of their performances. The conjugation of the sensing prototypes with the Internet of Things (IoT) for forming fully functioning sensorized systems is also shown here. Finally, some of the challenges existing within the current wearable systems are shown, along with possible remedies.

## 1. Introduction

The increase in the use of electrical and electronic equipment in daily life has improved the quality of life to a great extent. Compared with the past, the amount of time and energy required to finish a task while maintaining equal efficiency are much lower. In retrospect to the electronic industries, the use of sensing systems has exponentially increased in recent years [1,2,3]. These sensing systems have been produced and tested in the laboratory and in real-time situations. The popularization of sensing systems occurred in the 1990s, when semiconducting sensors were developed and utilized [4,5,6]. Single-crystal silicon [7,8] has been a prominent substance that has been highly preferred for forming the substrates of these sensors. Some of the advantages of single-crystal silicon sensors are the excellent signal-to-noise ratio, ability to work in harsh conditions, high stability and repeatability in the responses, and easy customization [9,10]. These silicon sensors have been fabricated using the microelectromechanical (MEMS) technique [11,12]. The attributes related to the MEMS technique are improved reproductivity, high accuracy, sensitivity, and selectivity. These silicon sensors have been used for different environmental [13,14] and industrial [15,16] applications. The materials used to form the electrodes mostly include nanoparticles, where certain metals, such as gold, platinum, and chromium, have been sputtered over the substrates to form thin-film layers [17,18]. Although these sensors have served a great purpose in sensing, some limitations still need to be addressed. For example, silicon sensor development for healthcare has been minimal compared with the other sectors. This has compelled researchers to develop flexible sensors. Flexible sensors have been developed using a range of polymers and nanomaterials, depending on their electrical, mechanical, and thermal characteristics [19,20]. Some of the common polymers that have been used to devise flexible sensors are polydimethylsiloxane (PDMS) [21,22,23], polyethene terephthalate (PET) [24,25,26], polyimide (PI) [27,28,29], and polyethylene naphthalate (PEN) [30,31,32]. Nowadays, conductive polymers, such as poly (3,4-ethylenedioxythiophene) polystyrene sulfonate (PEDOT: PSS) [33,34,35] and polyaniline [36,37,38], are used to develop nanocomposite-based flexible sensors [39,40,41]. Similar to polymers, certain types of nanomaterials, such as carbon-based allotropes [42,43,44] and metallic nanomaterials [45,46,47], have been used as processed materials to form sensors. The carbon allotropes include Carbon Nanotubes (CNTs) [48,49,50], graphene [51,52,53], and graphite [54,55,56], while some of the types of metallic nanomaterials are nanoparticles [57,58], nanoribbons [59,60], and nano-beads [61,62]. 

It can be seen that nanomaterials of all three dimensions are used to form sensors. After the mentioned polymers and nanomaterials were used to form flexible sensors, these prototypes were embedded with signal-conditioning circuits to form fully functional sensing systems. Flexible sensors have also been used for a wide range of healthcare [63,64,65], environmental [66,67,68], and industrial [69,70,71] applications. Among these uses, their deployment in the biomedical sector has been pivotal in recent years. Their utilization involves exploitation in two ways, including external attachment and implantation as implantable sensors [72]. Each method has its advantages, where the sensors adhere to their working principles for a particular application. Different kinds of wearable sensors, including tattoo-based [73,74,75,76,77,78], textile-based [79,80], and biofluidic-based [81,82] prototypes, have been implemented to date. These sensors have been used for personalized healthcare purposes, highlighting their efficacy in monitoring different diseases and the uninterrupted acquisition of data for a long time [83].

After the sensing prototypes were developed and characterized, they were embedded with electronic circuits to convert the sensed data into information. This part of the sensing system involves Internet of Things (IoT)-based devices for efficient data transmission. It is generally implemented by integrating sensors with customized printed circuit boards [84,85] or other microcontroller-based systems, where noise removal and data conversion occurs. The data are then transferred via different wireless protocols, such as Wi-Fi [86,87], Bluetooth [88,89], ZigBee [90,91] and radio-frequency identification tags (RFID) [92,93]. After the data are received at the monitoring unit, further analysis takes place to optimize the content of the data and classify them under appropriate channels. The use of this entire wearable sensing system for healthcare applications is reported in this paper. With the growth of medical technology and detection of new diseases, the need for wearable sensors for healthcare applications has constantly been growing over time. The simplified diagnosis of ailments using these sensors would help the patients to gain a better knowledge of their health status. Different kinds of prototypes are being developed that can be worn ubiquitously and at fixed intervals. These sensors are integrated with electronic conditioning circuits to form devices that can be used to determine the health information of a person [94]. In the current era of 3D printing technology, flexible sensors are being developed that can exhibit various exciting characteristics, such as being low cost and lightweight, and having high mechanical flexibility and high stretchability [95,96,97]. The electrode designs of these wearable sensors have been varied depending on the application of the wearable sensors. Some common electrode designs include interdigital, serpentine, and circular nature to assist the sensors in their use for resistive [98,99,100], capacitive [101,102,103], and impedance [104,105,106] measurements. The integration of software with wearable sensors helps in improving the health, quality of life, and quality of care. Another significance of wearable sensors is the need to protect the data of individuals. Different security protocols [107,108,109] have been applied over the years to the collected data to maintain the protection of the information of the patients. 

Even though researchers have previously presented the use of sensors for healthcare applications [110,111,112], this paper addresses some drawbacks. The novelty of this paper can be drawn in two aspects. Firstly, none of the review papers have presented a topical review of the description of an entire wearable sensing system, from fabrication to application. This paper encompasses the complete pyramid of wearable sensing systems formulated for biomedical applications. Secondly, none of the papers have classified the type of wearable sensors used for this application. Based on the nature of the sensors, two sub-classes of wearable sensors have been highlighted here. 

This paper is subdivided into twelve sub-categories. Followed by a brief introduction of the individual aspects of the paper, the sensors that are used on the market and research laboratories are elucidated in the second and third sections, respectively. These are followed by a detailed description of the use of IoT-based embedded systems, where the wireless technologies, data analytics for healthcare, customized IoT systems, cloud computing, and security issues are explained in sections four to nine, respectively. The challenges in the current designs, energy-harvesting issues, future possibilities, and, finally, the conclusion are drawn in the final section of the paper.

## 2. Healthcare and Wearable Devices (Product and Market Review)

Apart from the sensors fabricated and characterized in a controlled environment, some prototypes have been accepted and commercialized on the market. Many human societies have used these prototypes to deal with their anomalies. This section showcases some of the products available for the pivotal physiological ions and signals in the body. For example, certain companies, such as LifeScan, Hoffmann-La Roche, Medtronic, and others, are the leaders in generating digital glucose meters for monitoring diabetes [113]. These companies are expected to create more than 12 billion USD, with a compound annual growth rate of 21.7%, in the upcoming years. One advantage of these companies is that their presence in different countries can help the medical sector deal with the very high number of diabetes patients worldwide. Similarly, some of the leading companies that develop heart rate monitors include Polar, Sanitas, Zephyr, smartLAB, TRANSTEK, and others [114]. These companies can create devices that are capable of providing accurate heartbeats with an error of less than 0.5%. Although scientists across the world are trying to develop heart rate monitors with high sensitivity, robustness, and longevity, commercial companies have assisted in improving the quality of life by providing the instruments at reasonable prices. Another significant physiological parameter that is monitored using wearable sensors is blood pressure. Some companies, such as Hangzhou Enjoy Electronics & Instru, Andon Health Co., Ltd., Health and Life Co., Ltd., and Omron Dalian Co., Ltd., are some of the chief designers of blood pressure monitors. These point-of-care (PoC) devices allow people to perform measurements in a quick and non-invasive manner. Since strain sensing is a critical application for wearable sensors, companies, such as ATI Industrial Automatic, Morehouse Instrument Company, and TyTek Industries, should be mentioned as some of the significant manufacturers of efficient strain gauges. The recent ones are the implantable devices that are being fabricated worldwide. Abbott, Biotronik, Boston Scientific Corporation, Stryker, and Medtronic are some of the leading global companies that are the major players in the implantable medical devices market [115]. These companies have been able to generate a review of around 105.2 billion USD. Pharmaceutical companies are also gaining popularity in liaison with the electronic industries to design and develop wearable sensors. In this way, patients can obtain a direct subscription to the medication and its dosage after the anomaly is detected.

## 3. Sensors for Healthcare

The prototypes used for healthcare applications include a wide range of MEMS-based silicon [116,117,118] and printed flexible [119,120,121] sensors. Their morphological and chemical characteristics are designed in accordance with the application of the sensors. There are primarily two types of sensors that are used for healthcare applications. One of them consists of on-body sensors attached to different body organs of the human body. These on-body wearable sensors are attached to textiles for the comfortable monitoring of the targeted application. Some advantages of these on-body sensors are their simple attachment and non-invasive sensing performance. The second type of wearable sensor includes implantable prototypes placed inside the human body. They provide an effective way of monitoring the targeted application in real-time. The subsequent sub-sections involve some of the significant types of on-body and implantable sensors that have been developed and implemented in the healthcare sector. These examples showcase the exploitation of a wide range of polymers, nanomaterials, semiconducting single-crystals, and oxide-based alloys, where pure and nanocomposite-based sensors were formed for characterization and experimentation purposes. 

### 3.1. On-Body Sensors

The first category involving on-body sensors is the primary approach to detecting physiological signals from the body. Some of the on-body wearable sensor parameters include the detection of blood glucose, physiological signals, such as the heartbeat and respiration, and body motions. While each of these parameters plays a vital role in the human body, the approach of the on-body sensors used to detect them is different in terms of the working mechanism.

#### 3.1.1. Glucose Sensors

**Figure 1:**
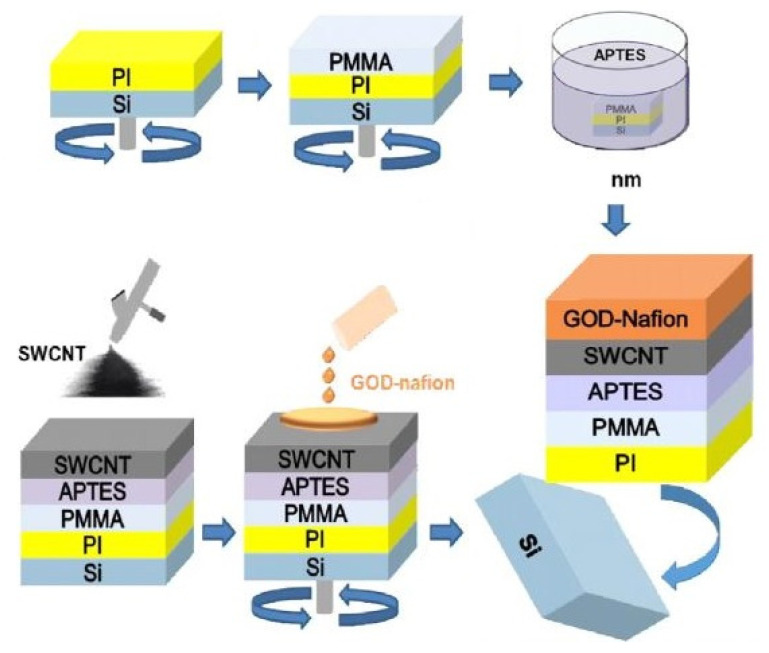
Schematic diagram of the fabrication process of SWCNT-based glucose sensors [123].

Müsse et al. [122] developed flexible enzymatic glucose electrochemical sensors using polystyrene (PS)–gold electrodes. The sensors were fabricated using PS foil with a thickness of 0.19 mm. The nanoimprinting process was used for fabricating electrodes with dimensions of 5 × 8 mm. The electrodes were finally formed using thin chromium and gold layers of 15 nm and 150 nm, respectively. A buffer solution was used for measuring purposes, where two values of 0.65 V and 0.7 V were considered as the measuring potentials. A linear range between 0.025 mM and 2 mM was obtained for an experimental concentration range between 0.025 mM and 25 mM. The sensitivity and limit of detection (LOD) of these sensors were 1.76 µA/mM/cm^2^ and 0.055 mM, respectively. Apart from the nanoparticles, carbon nanomaterials have also been used to develop highly sensitive wearable glucose sensors. Kang et al. [123] highlighted functionalized single-walled CNTs (SWCNTs) to develop glucose oxidase–Nafion composites for sensing purposes. Figure 1 [123] shows a schematic diagram of these SWCNTs-based flexible sensors. The SWCNTs were fabricated in different batches with a thickness of around 3–7 nm. The sensors could detect glucose at a level as low as 50 µM with a fast response time of less than 5 s. These prototypes could obtain a sensitivity of 41.397 µM^−1^ for a concentration range of 50 µM to 1 mM. They had excellent linear behaviour, with an R^2^ value of 0.99699. These sensors have been proven essential as low-cost and high-performance prototypes integrated with the user’s smartphone for diabetic patients. One of the interesting studies related to the development of glucose sensors is the work conducted by Zhao et al. [124]. Fully integrated and self-powered smartwatch sensors were formed using Zn–MnO_2_ batteries as intermediate energy storage units. 

The functionality of these prototypes was divided into three categories: glucose sensing, processing of the sensed signals, and displaying the results. Some individual units included in the system were photovoltaic cells, flexible batteries, a microcontroller, an E-ink display, glucose sensors, and a trans-impedance amplifier. The flexible batteries were formed by the pyrolysis of commercial melamine sponges on nitrogen-doped carbon foams. The flexible glucose sensors were fabricated on PET substrates. The electrode designs were developed using the photolithography process, followed by the thermal evaporation of chromium and gold on them. While the cyclic voltammetry process was used as the measurement process, the operation was carried out on printed circuit boards (PCBs). 

Similar to CNTs, graphene has also been used to develop low-cost electrochemical glucose sensors. Xuan et al. [125] showcased the development of nanocomposite-based sensors on flexible substrates. Reduced graphene oxide (rGO), used as the primary conductive material, was mixed with chitosan–glucose oxidase, gold, and platinum alloy nanoparticles. The micro-fabrication process was used to deposit these nanocomposites on the micro-patterns formed on polyimide substrates. The rGO was prepared using Hummer’s method and then reduced using a modified hydrothermal reduction process. The working electrodes of the prototypes were initially formed by the photolithography and wet etching processes. Finally, a spray-coating technique was employed to coat the working electrode with rGO aqueous solutions. The flexible polyimide polymers used to form the substrates were formed on a four-inch silicon wafer by a spin-coating process.

**Figure 2:**
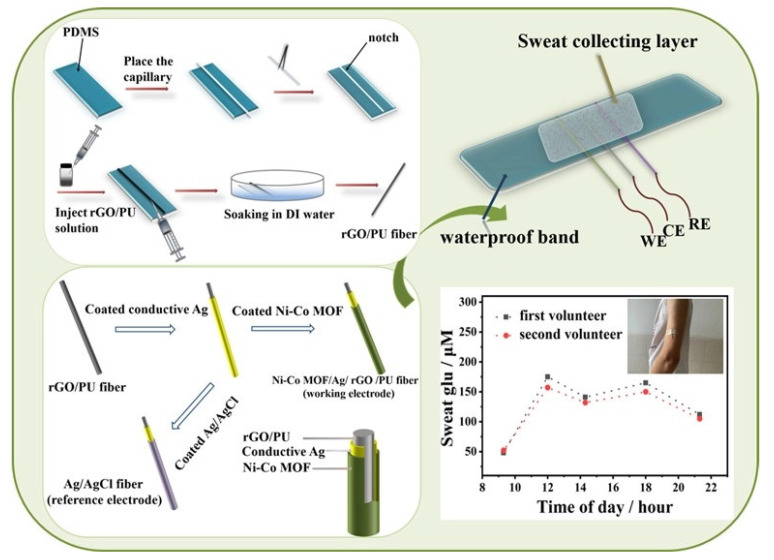
Fabrication and application of the Ni–Co MOF/Ag/rGO/PU fibre-based electrodes for the real-time monitoring of glucose molecules [127].

When used as wearable, sweat-based glucose sensors, the obtained detection range and linearity were 0–2.4 mM and 0.99, respectively. The sensors had a sensitivity of 48 µA/mM.cm^2^ and a response time of 20 s. The LOD of the sensors was 5 µm. Another example of nanocomposite-based wearable glucose sensors can be seen in the work conducted by Sedighi et al. [126]. Non-enzymatic sensors were formed using materials such as Ni–SnOx, polyaniline (PANI), and copper nanoparticles (CuO). The presence of nickel and copper oxide on the conductive templates had a strong influence in terms of the electrocatalytic activity to oxidize glucose. The coating of the PANI was conducted on phosphorus-doped nickel nanoparticles for the further deposition of CuO nanoparticles on conductive cotton surfaces. This enhanced the electron transfer acceleration between the electrodes and solution. The LOD of the sensors was 130 nM. The prototypes had sensitivities of 1625 µM^−1^.cm^−2^ and 1325 µM^−1^.cm^−2^ for concentration ranges of 0.001–1 mM and 1–10 mM, respectively. They also had a wide linear range of 0.001–10 mM. The prototypes also showed the excellent selectivity, reproducibility, and long-term stability of the responses for glucose-sensing applications. A similar example showing the use of nickel for developing flexible electrochemical sensors to detect sweat glucose can be seen in [127]. The advantages of these sensors are their large surface area and high catalytic activity. Highly stretchable sensors were formed by utilizing metallic organic frameworks (MOFs) of Ni–Co nanosheets, as shown in Figure 2 [127]. These MOFs were decorated using composite fibres consisting of silver, rGO, and polyurethane (PU). After synthesizing rGO/PU fibres using wet spinning technology, MOF nanosheets were coated on their surface to form the Ni–Co MOF/Ag/rGO/PU composites. The sensitivity and linear range of these sensors were 425.9 µA.mM^−1^.cm^−2^ and 10 µM–0.66 mM, respectively. The other attributes include the high stretchability and bending stability under different ranges of mechanical deformations, high accuracy, high selectivity, and long-term storage stability. When the electrodes were sewn on PDMS thin films, the three-electrode system showed efficient performance for the real-time monitoring of glucose molecules. 

The use of conjugated polymers for continuous glucose monitoring has been shown in the work conducted by Jin et al. [128]. Flexible, miniaturized, and fully integrated wearable systems were fabricated using screen-printed electrochemical biosensors. The support material was formed by using a biocompatible conjugated polymer, poly (N-phenyl glycine), for the immobilization of enzymes. The linear operating range of the sensors was increased by decorating the working electrode using a PU outer layer. The linear range and sensitivity of the sensors were 1–30 mM and 12.69 µA. mM^−1^.cm^−2^, respectively, during in vitro analysis. The sensors also showed long-term stability responses when the used buffer solutions were stored at a temperature of 4 °C. Yoon et al. also conducted in vivo analysis of the glucose molecules [129]. They developed wearable, robust, and non-enzymatic sensors using stainless-steel and electroplated nanoporous platinum-coated Nafion films. Some of the attributes of the sensors were high selectivity, reliability, fast response, and high sensitivity. The prototypes were tested with the interstitial fluid of rabbits at an interval of 5–15 min for detection purposes. The sensing system also consisted of an embedded conditioning circuit with a microcontroller unit and a wireless communication module. 

#### 3.1.2. Physiological Parameters

**Figure 3:**
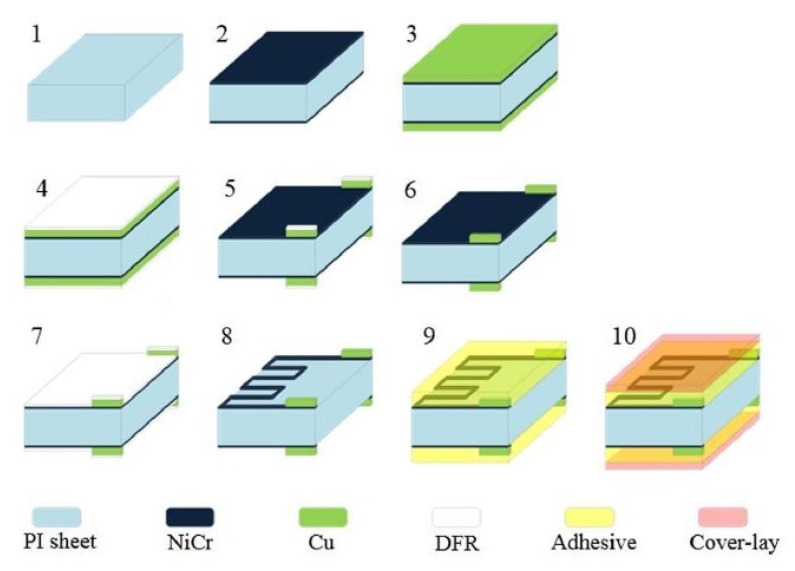
Schematic diagram of the fabrication steps of the Ni–Cr/Polyimide-based wearable heartbeat sensors [131].

The second category involves detecting pivotal physiological parameters of the human body. Some signals, such as heartbeat and respiration, were detected using low-cost, efficient wearable sensors. The performance of some of the efficient, flexible sensors that have been used as dry electrodes is mentioned in this section. Work on the fabrication of printed and flexible dry electrocardiogram (ECG) electrodes was reported in [130]. The use of these printed, flexible, and wearable dry electrodes to detect ECG signals assisted in performing the experiments without the need for any prior skin preparation using wet gel. The sensors were formed using the screen-printing technique, where silver flake ink was initially printed on PET substrates. This was followed by depositing composites formed by Multi-Walled Carbon Nanotubes (MWCNTs) and PDMS on them. The MWCNTs and PDMS were mixed with toluene and then coagulated at ratios of 1:12, 1:15, 1:25, and 1:50 to obtain weight percentages of 8%, 6%, 4%, and 2%, respectively. These conducting composites were deposited using a bar-coating process to obtain a thickness of around 284.7 ± 11.3 µm. The radii of these composite-based electrodes ranged between 8 mm and 16 mm. Finally, the samples were cured in the oven at 120 °C for 20 min. The sensors showed optimized performance in detecting the ECG signals for those with the most significant area. The sensors showed better results when the subject was in motion in terms of noise due to the better conformal contact at the electrode–skin interface. In contrast to carbon-based allotropes, Kwan et al. [131] reported the use of metallic nanoparticles to develop wearable, flexible sensors for heartbeat-sensing applications. Strain-gauge sensors were designed and developed using a double-sided fabrication method. Some of the advantages of these sensors include their high flexibility, compactness, and inexpensive nature. Figure 3 [131] illustrates a schematic diagram for fabricating these flexible strain gauges. Polyimide and nickel–chrome were used as the polymer and nanoparticles, respectively, to form sensors compatible with flexible printed circuit boards (FPCBs). 

The thicknesses of the sputtered nickel–chrome and electroplated copper on the polyimide substrates were around 0.04 µm and 13 µm, respectively. The electrodes had a line width of 70 µm and a line gap of 130 µm. The final step involved the attachment of the sensors to a silicone elastomer to enhance their elastic function and physical movements. The prototypes were presented as an array, with each prototype having a sensing area of 1.2 mm × 1.2 mm. The sensors displayed a linear response for a force of more than 930 kPa and a LOD of 6.25 Pa. The responses of the prototypes were linear for a bending radius of 5 mm to 100 mm. The recorded heartbeat response of the subject was around 14 beats per 10 s. 

The development of stable gel-less wearable ECG electrodes was also reported in [132], where healthcare patches were formed on flexible PET substrates. The thicknesses of the PET films chosen to create the substrates were 25, 50, and 100 µm, respectively. Silver was used to form the interconnecting electrodes. The samples were cured at 70 °C to form the top and bottom electrodes. These sensors were also capable of operating as temperature sensors. In order to function as ECG sensors, the adhesiveness of the prototypes was increased by using a mixture of ethoxylated polyethyleneimine (PEIE), MWCNTs, and PDMS, which also enhanced the electrical conductivity of the resultant prototypes. The number of CNTs and amount of PEIE mixed in the PDMS composites were around 10 wt. % and 3 wt. %, respectively. Finally, the samples containing PDMS-based conductive composites were cured at 70 °C. Another example related to the use of PET to form the temperature ECG sensors was reported by Yamamoto et al. [133]. Their work explained the fabrication and employment of all-printed, planar-type multifunctional wearable, flexible patches for acceleration, temperature, and ECG-sensing applications. The sensors were formed on PET substrates using CNTs, silver nanoparticles, and PEDOT: PSS as the conductive elements. The substrates were processed using laser cutting, mixing CNTs and silver nanoparticles to form the acceleration sensor. Then, the CNTs and PEDOT: PSS were mixed and printed to form the temperature sensors. Finally, the samples were cured at 70 °C, followed by the addition of the acrylic plate and silicone rubber as the mass and spacers, respectively. Three-electrode systems were used for the heartbeat measurement. 

**Figure 4:**
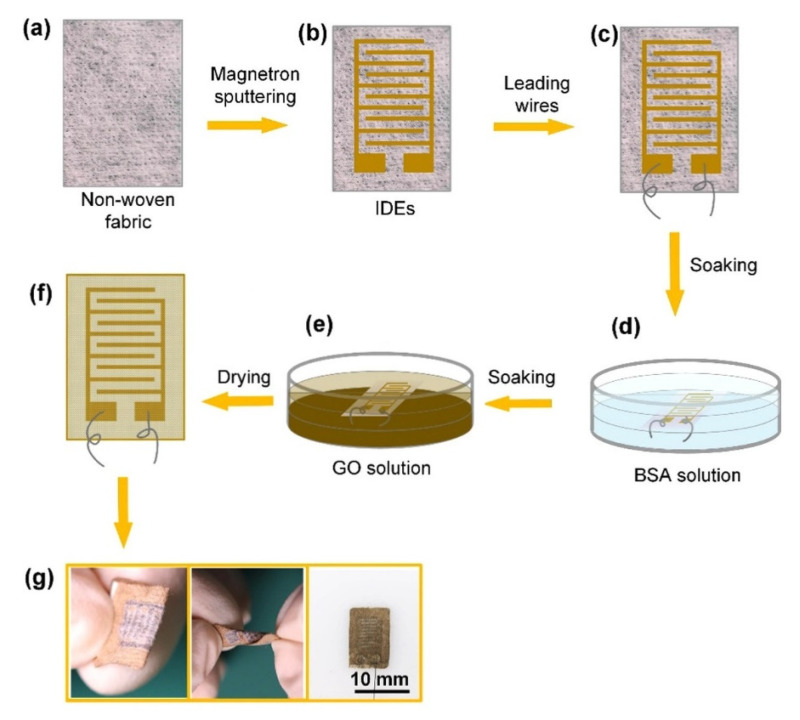
Representation of the fabrication steps of the GO/non-woven fabric-based sensors for respiratory applications. (**a**) The non-woven fabric taken as the processed material was used as substrates to form (**b**) interdigitated electrodes (IDEs) and (**c**) attach connecting wires. (**d**) The samples were then treated with (**d**) bovine serum albumin (BSA) solution and (**e**) graphene oxide (GO) solution. (**f**) The samples were then dried and used for experimental purposes. (**g**) The mechanical flexibility and final dimension of the sensors [134].

Similar to heartbeat monitoring, the detection of respiration was also conducted by using these types of wearable sensors. Wang et al. [134] illustrated the development of highly sensitive wearable and flexible sensors for respiration-monitoring applications. These sensors consisted of a non-woven fabric (NWF) coated with graphene oxide (GO) to form the sensing area. The adsorption of GO on NWF was enhanced by using bovine serum albumin. Figure 4 [134] presents the fabrication steps of these wearable sensors. While interdigitated electrodes were chosen as the design, the magnetic sputtering of chromium and gold was conducted to form the electrical connections. The line width and interdigital gap of the electrodes were 500 µm and 550 µm, respectively. The electrodes were then filled using conductive silver adhesives and cured at 120 °C for half an hour. Then, the sensors were soaked with BSA solution and cleaned using deionized (DI) water. This was followed by depositing GO on the sensing area prepared by dispersing commercial GO nanosheets in DI water. Each sensor had dimensions of 12 mm × 10 mm × 0.2 mm. These sensors were tested under different conditions, including fast and deep breathing, nose and mouth breathing, and identification of spoken words. 

Another aspect of this application is shown in the work conducted by Park et al. [135], where flexible, capacitive pressure sensors were developed for monitoring respiration. Here, porous Ecoflex with a porosity of 36% was used to form the dielectric layer of the capacitive pressure sensors. This layer was developed by the processing of a sugar cube via a simple melting process. The prototypes also consisted of PDMS, silver nanowire, and carbon fibres as the processing materials. The carbon fibres had a diameter of 7 µm and a length ranging from 1–2 mm. The carbon fibres were initially used to form dispersions by mixing them with dimethylformamide, which was poured into poly (methyl methacrylate) moulds formed on silicon wafers. These dispersions were then cured at 100 °C for half an hour. Then, silver nanowires were poured onto the samples and spin-coating was carried out. Finally, the silicon wafers were treated with a 10% sodium chloride solution and dried in the oven at 95 °C. These sensors exhibited a high sensitivity of 0.161 kPa^−1^ and excellent durability of over 6000 cycles. The working pressure range was also high, showcasing enhanced responses for pressures lower than 10 kPa and higher than 200 kPa. Experiments were carried out by integrating the sensors into clothes. The final experimental step involved attaching these sensors to a waist belt for the real-time monitoring of the respiration signal of human beings. 

**Figure 5:**
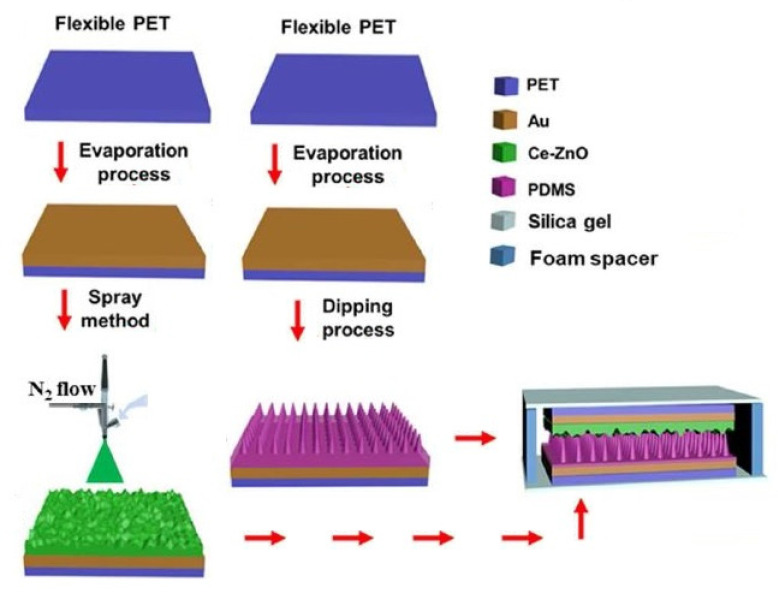
Illustration of the fabrication steps of the PET/PDMS/Silica gel/Ce-ZnO-based wearable respiration sensors [136].

Jiang et al. [136] reported interesting work highlighting the development of integrated flexible, self-powered, and wearable respiration sensors. The triboelectric sensors contained a specific amount of doped Cerium (III). Figure 5 [136] demonstrates the fabrication steps of these triboelectric, wearable respiration sensors. The sensors were formed using PET polymers and gold nanoparticles to form the polymers and electrodes, respectively. Zinc oxide (ZnO) nanocomposites were formed by doping with cerium at different ratios from 0–0.01 M. The final optimized Ce-doped value of the ZnO nanocomposites was 0.004 M. PDMS was used as the triboelectric layer and was modified using the template method. The two layers, Ce-doped ZnO and PDMS, were affixed using two soft silica gel films with 100 µm thickness. The final step included packing the device using a flexible sealing film to generate a total thickness of 3 mm. Some of the primary attributes of these prototypes were their high selectivity and high sensitivity of 20.13 ppm^−1^. After the sensors were triggered by the expansion and contraction of the chest during the breathing process, these sensors could simultaneously detect the human respiratory patterns and physiological processes after intensive motions.

One of the very interesting studies conducted by Presti et al. [137] showcases the development of wearable systems capable of monitoring both heartbeat and respiration. These strain sensors were based on Fibre Bragg Gratings (FBGs) coupled with polymers. These flexible sensors were encapsulated into Dragon skin 20 silicone rubber before obtaining their responses towards strain, template, and relative humidity. The two parts of silicone rubber were mixed, degassed, and then poured on customized 3D-printed moulds. After the FBG was placed in these moulds at the midsection, dragon skin vulcanization was performed at room temperature for four hours. The final dimensions of these FBG-based sensors were 90 mm × 24 mm × 1 mm. During the respiratory test, the upper and lower cut-off frequencies of the Butterworth filter of the signal-conditioning circuit were 0.8 Hz and 2 Hz, respectively. The sensitivity of these sensors towards the applied strain was 0.125 nm.mε^−1^. The sensors also showed a linear response with a high R^2^ value of 0.994.

#### 3.1.3. Body Movements

**Figure 6:**
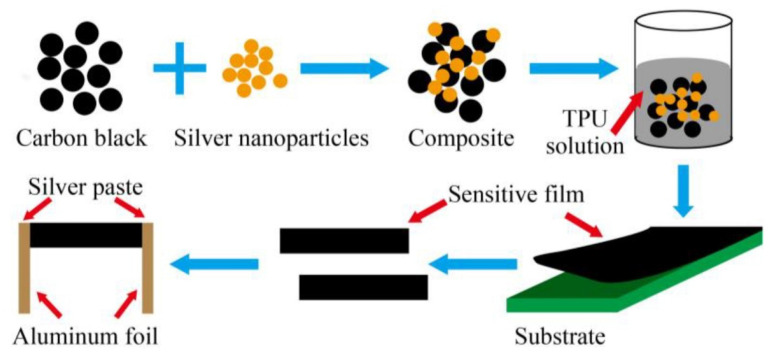
Schematic illustration of the fabrication process of the carbon black/silver nanoparticles/ thermoplastic PU nanocomposites-based strain sensors [138].

The subsequent pivotal use of wearable sensors for healthcare applications involves detecting physiological movements. Wearable sensors have been used to monitor the minor and significant movements of different body parts. Zhang et al. [138] elucidated the fabrication of nanocomposites-based flexible strain sensors for human motion detection. These nanocomposites were formed using a mixture of carbon black and silver nanoparticles at defined ratios. A simple, low-cost, and convenient strategy was used to develop these prototypes. The conductive nanomaterials were mixed in thermoplastic PU as the polymer matrix. Figure 6 [138] illustrates the fabrication process of these wearable, flexible strain sensors. Initially, the surface of the carbon black nanoparticles was modified by a poly (vinylpyrrolidone) grafting process. Then, homogenous solutions were formed using carbon black, followed by forming the nanocomposites by adding the silver nanoparticles with vigorous stirring. The mass ratio between the carbon black and silver nanoparticles was around 3:1. The conductive composites were then added to the TPU by mixing them at different mass fractions ranging between 2 and 50 wt. %. Some of the attributes of the sensors were their high stretchability, high sensitivity, and excellent static and dynamic stability. The prototypes had a gauge factor (G.F.) of 21.12 at 100% tensile strain and showed stability in their responses for over 100 cycles. These nanocomposite-based strain sensors had responses 18 times better than those of sensors formed using bare carbon black. The prototypes detected certain human motions, such as finger bending, wrist rotation, and elbow flexion.

Another study highlighting the use of carbon black for forming flexible strain sensors was reported in [139]. A facile, effective, low-cost, and convenient strategy was used to process the ZnO nanorods, carbon black, and PDMS as the raw materials. The advantages of these sensors are their high mechanical flexibility, high sensitivity, large workable strain range, good linearity, and ideal stability. The ZnO nanorods were synthesized by mixing zinc acetate and methenamine in equal amounts to form homogeneous solutions in a Teflon-lined reaction vessel. The solutions were treated with stirring and centrifugation techniques at elevated temperatures. Finally, the nanocomposites were formed by adding ZnO/carbon black to the PDMS matrix and treating with an ultra-sonication process. The final contents of ZnO nanorods in the composites were 1 wt. % and 4 wt. %. Guan et al. [140] showcased a significant study where wearable strain sensors for monitoring human movements were developed using casein-driven adhesive and anti-freezing hydrogels. Casein and lithium chloride were introduced into the hydrogel to develop a tough and adhesive PAAm/Casein hydrogel. Each of these elements was considered at different ratios. These mixtures were poured into glass moulds with the presence of silicone spacers. The samples were then cured at 40 °C for four hours to obtain the final product. These sensors exhibited excellent mechanical properties and excellent reversible adhesive behaviour in human skin and other related materials. The highest conductivity and anti-freezing properties of these composites were 0.0753 S/cm and −21 °C, respectively. These prototypes were able to detect both large-scale and minuscule human movements.

Similar work was reported in [141], which showed the design and development of flexible, adhesive, and self-healable hydrogel-based wearable strain sensors for monitoring human motions and physiological signals. Multifunctional conductive hydrogels were formed that comprised a polyacrylamide and chitosan hybrid network. The crosslinking of this network was further developed by mixing them with carboxyl functionalized MWCNTs at defined amounts. The nanocomposites, after formation, were transferred to customized moulds that consisted of two parallel glass plates and a silicone spacer. The samples were then cured at 45 °C for six hours to form hybrid network hydrogels. Certain advantages of these sensors included their good flexibility, puncture resistance, and self-healing capability. The hydrogels properly adhered to different materials, such as wood, glass, aluminium, rubber, polytetrafluoroethylene, and skin. The applications of these sensors included some human activities, such as joint motions, speaking, and subtle blood pulse. These sensors also showed excellent durability for certain applications, such as artificial intelligence, soft robots, biometric prostheses, and health-monitoring systems.

Another example related to using hydrogels for monitoring human motions can be seen in the work conducted by Xu et al. [142]. Adhesive, tough, and self-healing hydrogels were used to form flexible strain and pressure sensors. Casein sodium salt (SC) from bovine milk and polydopamine (PDA) were mixed with polyacrylamide hydrogel systems to form the resultant prototypes. The SC-PDA hydrogels were prepared using a simple free radical polymerization process. The SC and PDA were mixed and injected into a reaction mould with a thickness of 6 mm. These moulds were formed using a pair of glass plates and silica gel. Some of the attributes of these sensors were their super-stretchable nature and excellent fatigue resistance. The presence of sodium ions assisted in sensitive deformation with respect to the conductivity of the SC-PDA hydrogels. These sensors were used to detect large-scale motions, such as bending joints, and tiny physiological signals, such as speaking and breathing. The bending of joints included jumping and slow and fast walking. 

Graphene has also been extensively used to develop wearable sensors to detect human movements. Wang et al. [143] presented the use of graphene woven fabrics (GWFs) to develop highly sensitive strain sensors for monitoring human motions. These GWFs were adhered to polymer and medical tape composite films to achieve certain characteristics, such as an ultralight weight, high sensitivity, high reversibility, and excellent robustness. The easy fabrication steps of these sensors allowed them to be used for detecting changes in resistance for certain motions, such as hand clenching, phonation, expressions, blinking, breathing, and pulse rate. Another use of graphene can be seen in the work conducted by Sun et al. [144], where wearable, waterproof, and highly sensitive strain sensors were developed for the wireless monitoring of human motions. The electrodes of these sensors were formed using 3D graphene, carbon black, and nickel sponge. A simple, cost-effective, and scalable drop-coating method combined these nanomaterials with PDMS. Initially, two copper wires were connected to the nickel sponge with silver paste to form the electrodes. Then, suspensions were formed using graphene, carbon black, and PDMS and dropped on the nickel sponges to form the resultant prototypes. The skeleton of the nickel sponge then anchored to that of the drop-casted graphene and carbon black. Due to the presence of PDMS, the sensors were hydrophobic and had a water contact angle of 129°. These strain sensors developed excellent flexibility, long-term stability, and a high sensitivity of 138 at 16% strain. A wireless sensing system was formed using the fabrication sensor, a STEM32 controller, a ZigBee module, and a portable power source. The prototypes could detect subtle human motions, such as pulsing, blinking, and swallowing. One of the interesting studies showcasing the use of MEMS-based sensors to detect human motions was reported in [145]. Multifunctional, wearable piezoelectric MEMS sensors were developed for monitoring motions, health warnings, and earphones. The inverse piezoelectric effect was carried out using piezoelectric thin films (PZTs). These PZTs were fixed in a rigid–flexible coupling sealed vibration membrane to minimize acoustic loss and avoid airflow loss. Silicon was used to form the substrates with a silicon dioxide layer as the silicon-on-insulator for these multi-layered structures. Parylene C was used as a thin membrane on the surface of the MEMS-based sensors. The size of these sensors was less than 10 mm. When an input voltage of 2 V was applied, the sensors could obtain SPL higher than 55 dB for a full frequency range. 

### 3.2. Implanted Sensors

Implantable prototypes for healthcare applications are the second category of wearable sensing prototypes. These sensors have been inserted inside the human body to detect anomalies or deliver micro-level drugs. These sensors provide effective real-time monitoring of the changes inside the body. They provide a straightforward manner to transport molecules and monitor vital signs. Two categories, including neural and drug-delivery sensors, have been considered to perform as implantable sensors for operating in the healthcare sector. 

#### 3.2.1. Neural Sensors

Lee et al. [146] illustrated the development of battery-free triboelectric nanogenerators (TENGs) for operating as a neural interface and modulated the control of tibialis anterior muscle via peroneal nerves. These devices functioning in a battery-free manner obtained an output voltage and short circuit of 160 °C and 6.7 µA, respectively. These flexible and adjustable neural interfaces could function as flexible sling electrodes. A direct simulation was performed on the sciatic and common peroneal nerves in rats. Multi-layered, stacked designs were formed that contained a PET sheet for a zigzag-shaped structure. These structures provided both space, assembly, and recovery force after pressing. The PET layer was attached to copper films to form two pairs of contact layers. Finally, the bottom part of the copper electrodes for each pair was attached to PDMS films consisting of micro-pyramid patterns. Optimization was conducted on different lengths of PET films to encompass the TENG devices. The MEMS technique was used to fabricate the neural interfaces. These interfaces were sling-shaped with two layers of polyimide and gold sandwiched between them. 

Another work related to the use of TENG in neural sensing applications was reported by Zheng et al. [147]. Biodegradable TENG was used as a lifetime-designed implantable power source for neural repairs. The fabrication process of the TENG started by forming solutions using poly (L-lactide-co-glycolide) (PLGA) and chloroform, and casting them on a glass plate. The solutions were then dried and treated with other chemicals before sputter-depositing a layer of magnesium on the flat side of the resultant thin films. The two thin films were then attached with a spacer between them. Finally, two lead wires were attached to the thin films to form the electrodes. The devices were also formed using other polymers, such as poly (vinyl alcohol) and poly (caprolactone) (PCL). The thickness of the films was controlled to 100 µm. The TENG devices were then encapsulated using PLGA polymer via the casting method. Then, the thin films were cut to appropriate sizes and the edges of the two layers were attached using PLGA solutions. Finally, the devices were air-dried and sealed using a heat sealer. 

**Figure 7:**
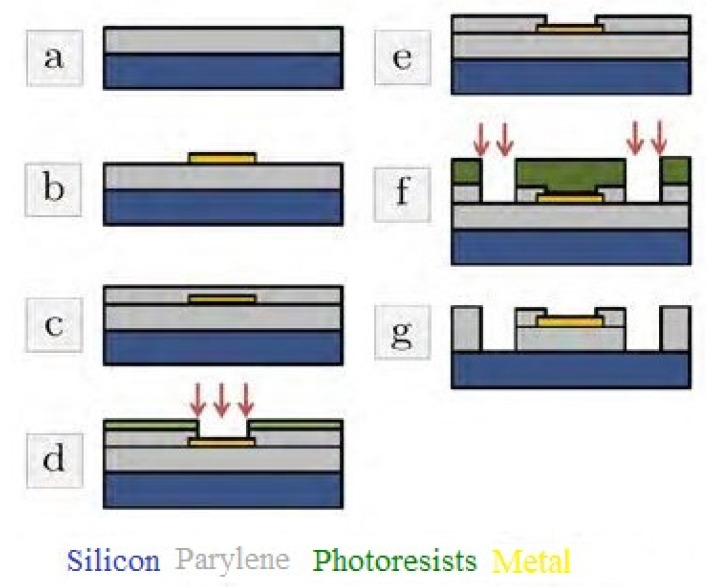
Schematic diagram of the fabrication process of the Parylene C/PEDOT-based neural sensors. (**a**) The deposition and annealing processes of Parlyene (**b**) Metallization and lift-off processes of the metallic layers were carried out. (**c**) Passivation process with Parylene C and annealing. (**d**) Passivation plasma etching process was carried out for electrode opening. Other processes like (**e**–**g**) plasma etching and photoresist stripping [149].

Murthy et al. [148] elucidated the design and simulation of implantable blood pressure sensors using COMSOL Multiphysics. The nanotube-based sensors were developed using the MEMS technique and were used to determine the change in displacement with respect to pressure. The performances of these sensors were compared to those of blood pressure sensors developed using silicon and gold. The pressure range chosen for the simulation was fixed between 5 and 11 kPa. One of the interesting studies, as explained by Lecomte et al., was reported in [149], wherein in vitro and in vivo bio-stability analysis was conducted for chronically implanted Parylene C neural sensors. These neural probes consist of PEDOT-nanostructured gold structures used for recording brain activities. Figure 7 [149] illustrates the fabrication process of these MEMS-based neural sensors. The implant consisted of gold patterns that were placed in a sandwiched manner between two Parylene C layers. The thicknesses of the two layers were 23 µm and 1.5 µm. Then, annealing of the layers was conducted at 100 °C for sixteen hours to improve the adhesiveness of the layers. Then, deep plasma etching was conducted on the implants to cut through the 25 µm layers, followed by attaching four recording microelectrodes. These electrodes had a diameter, length, and width of 40 µm, 2 mm, and 250 µm, respectively. This was followed by performing the potentiodynamic route on the electrodes using the PEDOT: PSS solution. The shank was then treated with a silk fibroin coating process that was gutter-shaped and had a maximum dimension of 130 µm. The degradation of the silk–fibroin was treated with a water-annealing process to modify the inherent structure. In vitro and in vivo impendence measurements showed that the soaked devices were stable and had 10% of their initial values in the artificial cerebro-spinal fluid. Testing was conducted for six months at a temperature of 37 °C. The sensors also showed a steady signal-to-noise ratio (SNR) and accurate wireless measurement of high-amplitude hippocampal local field potentials. The sensors responded reliably to artificial and actual physiological conditions. 

Another Study explaining the use of PEDOT: PSS for neural sensors was reported in [150]. Durable, soft microelectrodes were developed using composites that consisted of PEDOT: PSS and GO as processing materials. After mixing PEDOT: PSS and GO to form hybrids, they were electrochemically deposited on gold microelectrodes. The variation in the redox states, composition of the GO, and the electrochemical performance of the composites simultaneously varied in neural sensors. After GO was synthesized via the oxidation of graphite powder in accordance with the modified Hummers method, it was reduced using hydrochloric acid. Then, the composites were formed by adding the polymers PEDOT and PSS at a weight ratio of 1:1. Finally, gold wire electrodes were formed by depositing PEDOT: PSS/GO composites on gold wire and sealing them with epoxy. The penultimate step included curing the coated gold wires in the oven at 50 °C for a couple of hours, followed by cutting and polishing using sandpapers. The changes in the responses were calculated in terms of the current at different voltages. 

Another interesting study related to the implementation of wearable neural sensors can be seen in the work conducted by Hurtado et al. [151]. Their paper showcases the development of flexible micro-displacement sensors for wearable, implantable biomedical applications. These sensors consisted of micro-fabricated sliding interdigitated gold plates and a circular coil. The primary core of the sensor consisted of flexible substrates. A wireless, passive detection scheme was used to study the dynamics of an implanted abdominal mesh. The resonant frequency of the sensors was determined with respect to the sliding magnitude, which eventually decided the resultant capacitance. Cyclo-olefin polymer was used as the dielectric layer in varying thicknesses to analyse the simultaneous changes in these sensors. While ex vivo characterization was conducted to study the behaviour of the tissues, the obtained sensitivities were 0.04 pF/µm and 0.85 pF/µm for a displacement range of 0–600 µm. The maximum sensitivity and resolution of the sensors were 410 pF/µm and 1 µm, respectively. The sensors could operate with a frequency shift up to a maximum limit of 20 mm and 10 mm in air and phantom, respectively. CNTs have also been considered to develop wearable neural sensors, as reported in [152]. Soft peripheral nerve interfaces were made from CNTs embedded in silicone polymers. CNTs were embedded in PDMS using a selective vacuum filtration process via printed wax patterns. Initially, filter membranes with a pore size of 0.22 µm were patterned with negative structures of the electrode designs using a wax printer. Then, CNTs were mixed with surfactant and DI water to form homogenous solutions with a pH between 5 and 7. The CNTs were then filtered and dispersed on the target electrodes, followed by dipping the membranes in acetone four times. Two PDMS layers were formed on glass slides by the spin-coating technique. The membranes were then dried and pressured onto a semi-cured PDMS layer that was formed by curing the mixed silicone at 65 °C. Another PDMS layer was formed by fully curing it at 100 °C. Finally, the membranes were washed again with DI water and peeled off to leave the CNTs’ patterns on the PDMS surface. The sensors showed excellent mechanical and electrochemical stabilities towards the stretching cycles and strain-related characterization techniques. The simulation process was carried out for the central nerve cord of a horse leach to determine the capability of these interfaces within the window of the CNTs/PDMS electrodes.

#### 3.2.2. Drug-Delivery Sensors

**Figure 8:**
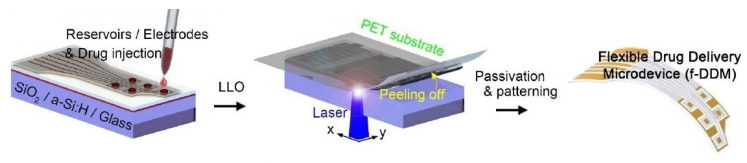
Illustration of the fabrication of flexible drug delivery micro-devices [153].

The next category of implantable sensing prototypes involves drug-delivery sensors. These sensors can transport essential molecules over a wide range of concentrations. These sensors have been employed in different biosensing applications, where continuous monitoring can detect acute and chronic diseases. Sung et al. [153] highlighted the development of a flexible, wireless powered drug delivery system for targeted administration on the cerebral cortex. Flexible drug delivery micro-devices consisting of freestanding gold membranes over the multi-reservoir array were formed and implemented via opting for a reverse fabrication process of the reservoir and sealing membrane. Figure 8 [153] represents the fabrication steps of these flexible drug delivery micro-devices. Exfoliated hydrogenated amorphous silicon and silicon dioxide layers with thicknesses of 50 nm and 1.5 µm, respectively, were used as the exfoliation and buffer layers. These two layers were deposited on rigid substrates using an enhanced chemical-vapour-deposition process. The sensor consisted of a PET substrate, a multi-reservoir array, a metal sealing membrane, and a passivation layer. Two layers of titanium and one sandwiched gold layer with thicknesses of 20 nm and 100 nm, respectively, were deposited using an electron beam evaporator and conventional lithography processes. Multi-reservoir arrays were formed using SU-8 photoresist and bonded on flexible PET substrates using an inorganic laser lift-off process. The thickness of the PET substrates was 25 µm. The surface area and thickness of the micro-reservoir arrays were 300 × 300 µm^2^ and 15 µm, respectively. The electrodes and sealing membrane of the micro-reservoir were formed using the metal layer. Finally, the device was passivated by SU-8, and wet etching was conducted on the silicon dioxide and titanium layers to open the electrodes. The optimization of the design of these micro-devices was conducted using finite element analysis to minimize thermal damage during laser transfer. A stable, wireless powered operation was carried out to successfully deliver two different chemicals and use an anti-epileptic drug to prevent seizure activity. 

One of the interesting studies showing the deployment of a dermal patch with integrated, flexible heaters for drug delivery applications was reported in [154]. The fabrication process included the formation of thermo-responsive drug micro-carriers encapsulated with a hydrogel layer. The attachment of the heaters to these sensors was conducted using integrated electronic heater control circuitry. The hydrogel patches were formed by mixing sodium alginate and distilled water at a temperature of 4 °C. The crosslinking process was carried out using a calcium chloride solution and agarose. Then, the solution was poured into a mould formed using PDMS and solidified at room temperature. The last steps included peeling off the agarose sheet, moving the solution to another PDMS mould, and using them to cover the sodium alginate to form calcium alginate sheets. The capabilities of these devices include conformal covering of a wound area, controlling the delivery of drugs, and adjusting a hydrogel layer. The release rates of the drugs could be controlled by encapsulating them inside micro-particles. Microfluidic devices were used to fabricate these active molecule-containing monodisperse thermos-response micro-particles. Two different active molecules that had molecular weights similar to the drugs and growth factors were released and characterized. 

Lee et al. [155] reported the design and development of flexible, sticky, and biodegradable wireless devices for drug delivery to brain tumours. Spin-coating of poly (methyl methacrylate) (PMMA) was conducted on a silicon wafer to use as a sacrificial layer. After this layer was cured at 180 °C for a couple of minutes, polyimide precursor solutions were spin-coated on the substrates and cured at 250 °C for a couple of hours. Then, zinc oxide thin films were formed as an adhesion layer by using the AC sputtering technique under an argon atmosphere. This was followed by the spin-coating and patterning of the photoresist. Then, magnesium was deposited and etched to form a wireless heater and temperature sensor. The two polyimide layers were then patterned via reactive-ion etching, followed by etching the PMMA layer using acetone. The final steps involved picking up the device using a PDMS stamp and etching the lower polyimide layer. Then, polyvinyl alcohol was spin-coated on the film and the two-layer polyimide layer using an oxygen plasma process. In the end, the encapsulation of these magnesium-based devices was conducted using poly (lactic acid) and poly D, L-lactic-co-glycolic acid polymers. Drug-loaded patches were integrated with wireless electronics and deployed for controlled intracranial drug-delivery applications. The mild thermic actuation process was used by these bi-facially designed sticky/hydrophobic devices for conformal adhesion to the brain surgery sites. These patches could control the drug leakage to the cerebrospinal fluid by using spatially controlled and temporarily extended drug techniques. The neurological side-effects were also minimal due to the biodegradation of the entire device. They were used in a mouse model to confirm the tumour volume suppression and improved survival rate. Another example of flexible, biodegradable sensors can be seen in [156], where flexible nanofibrous platforms were formed with integrated, flexible heaters for on-demand drug delivery. Thermally controlled, antibiotic-releasing nanofibrous sheets were created via the electrospinning of poly (glycerol sebacate)–poly (caprolactone) (PGS-PCL) to fabricate elastic polymeric sheets. The diameters of these fibres ranged between 350 nm and 1100 nm. The substrates were flexible in nature, with a tensile modulus of around 4–8 MPa. PEGylated chitosan and the nanoparticles were separately prepared before assembly, characterization, and experimentation. 

PGS was synthesized using a poly-condensation process and sebacic acid and glycerol as the processing materials. The temperature and pressure were fixed at 120 °C and 40 more, respectively, for 24 and 48 h. The direct patterning of bio-resorbable metallic heaters was conducted on the nanofibrous substrates to execute thermal stimulation for releasing the antibiotics on demand. These devices showed high biocompatibility and biodegradability during the in vitro studies.

Feng et al. [157] elucidated the fabrication and implementation of piezoelectric devices for developing flexible and stretchable electronics for multifunctional applications. These devices were used for monitoring knee health and enhanced drug delivery applications. The sensors were based on thin polymer poly (N-isopropyl acrylamide) (PNIPAM) films as they were stimuli-responsive. The packaging of these sensors was conducted in PDMS to form smart patches. The smart materials included piezoelectric lead zirconate titanate (PZT) and thermally activated poly (N-isopropyl acrylamide) in operating sensing acoustic emission waves and drug-delivery applications, respectively. The titanium sheet had a 50 µm thickness and was processed using the standard photolithography technique. The growth of the PZT was conducted using the hydrothermal process at 180 °C. The PNIPAM gel was used to develop the thermal actuator and drug-releasing element. It consisted of using a shadow mask, allowing the sputtering of a 1 µm silver film on one side of the PNIPAM element. A 3D printing process was employed to develop the moulds that could form and fit the PNIPAM gel in a circular shape. Thin, flexible titanium sheets were used to form the arrays in the shape of an electrically connected serpentine network. The flexible sensors were embedded in a knee-band strap to analyse the acoustic emission waves released during certain activities, such as hopping and jumping. 

**Figure 9:**
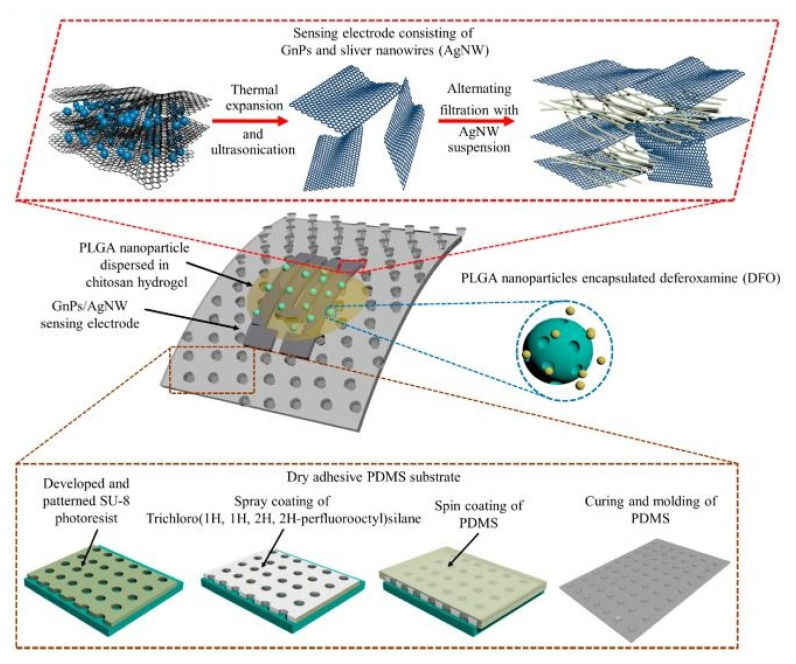
Illustration of the different components present in the strain-sensing and drug-delivery sensors [158].

The nanocomposites have also been used to develop drug wearable -delivery sensors, as shown in [158]. Multifunctional prototypes were formed using nanocomposites that contained graphene and silver nanowires as conducting nanomaterials. These sensors were employed for highly sensitive-strain sensing and drug-delivery applications. A simple and cost-effective technique was used to develop the sensors with certain components, such as skin-mountable dry adhesive substrates, robust sensing elements, and a transdermal drug delivery system. Figure 9 [158] shows an illustration of these components. The substrates of these sensors were formed using PDMS micropillar arrays on five-inch silicon wafers. These PDMS arrays had a diameter of 10 µm and an aspect ratio of 3:1. The pull-off strength of these micropillar films was around 1 N/cm^2^. A filtration-patterning technique was used to process the nanocomposites to obtain a thickness of around 40 µm. Sandwich-structured geometry was formed on these nanocomposites on PVDF films. The effective sensing area was around 5 cm^2^, and was able to adhere to the silicon wafer and human arm. The drug-delivery mechanism comprised of polylactic-co-glycolic acid nanoparticles and a chitosan matrix. The nanoparticles were released into the stratum corneum up to a depth of 60 µm.

One interesting study on wearable drug delivery systems was reported in [159], where flexible devices were developed for ocular iontophoretic drug applications. The sensors were deployed for in vivo experiments on rabbit eyes, where they were placed under the eyelid. This process was carried out by reducing the tissue damage to deliver ions through a small area on the eyeball. When the device was placed on the eyelid during iontophoresis, manganese ions were delivered as a tracer to reduce the optic nerve damage in the rabbit eyes. For 600 s, a 102 ng/mL concentration during iontophoresis was delivered compared with the average concentration of 271 ng/mL. A wide pulsed current under a frequency of 1 kHz was applied as the current–voltage characteristic. The local temperature during the operation was 38 °C and the applied current was less than 2 mA. Rajabi et al. [160] explained the development of flexible, stretchable microneedle patches for transdermal bio-interfacing applications. These devices integrated soft and flexible base substrates with rigid, stainless microneedles. The elastomeric polymers assisted in the robustness and conformal contact between the elements. The magnetic assembling of short stainless-steel microneedles was exploited and deployed on the surface of the human skin. Polystyrene film was used to form an array of 7 × 7 holes, with each one having a diameter of 220 µm and a pitch of 2 mm. The polymer was then cut into an active area of 18 × 18 mm^2^ using an assembly process. PMMA was used as the template to cover the top surface of the microarrays with a laminated release liner. The thickness and size of the de-moulded substrates were 100 µm and 20 × 20 mm^2^, respectively.

## 4. Wireless Technologies for the Wearable Healthcare System

**Figure 10:**
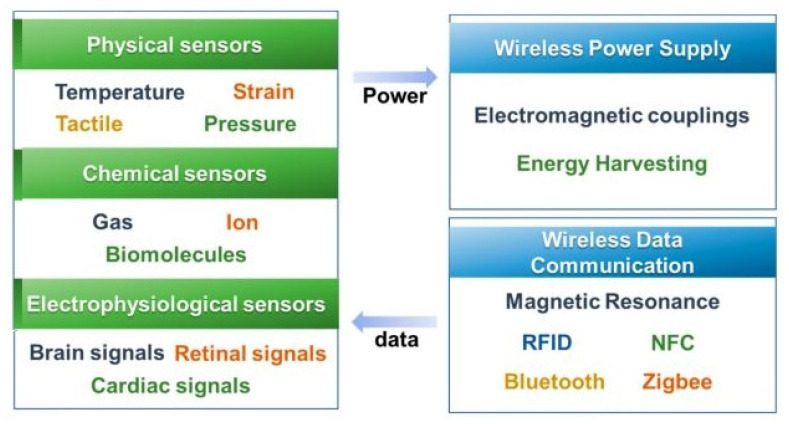
Representation of the components of the wireless protocols used for wearable healthcare systems [161].

The wireless protocols used for wearable sensors significantly vary depending on their intrinsic properties. The dynamicity of wearable sensors used in households and controlled environments varies based on the detected physiological signal type. Various types of sensing networks are being used to design the architecture for wearable sensing applications. Some of the attributes of these networks, such as cost, configuration, and power consumption, are considered when integrating with wearable sensors. Figure 10 [161] shows a schematic diagram of some protocols embedded with physical, chemical, and electrophysiological sensors. In order to operate the sensing prototypes, the use of radio-frequency identification tags (RFID), Bluetooth (IEEE 802.15.1), Wi-Fi (IEEE 802.11), and ZigBee (IEEE 802.15.4) protocols have been considered for wireless power supply. The range of these protocols varies from 10 m to a few kilometres. The efficiency of these protocols is around 60–80%, based on the routers connected to a single network. The variation in the frequencies used for each protocol is also significant, from a few kilohertz to a few gigahertz. Using batteries without power transfer techniques allows the prototypes to be used ubiquitously for more extended periods. 

They allow remote data communication, especially for detecting the signals of patients and elderly people. Table 1 [162] provides an overview of some of the communication protocols used for operating wearable sensors. The energy harvesting phenomenon has also been studied to operate wearable sensors by using energy harvesting devices both as power supply and sensing devices. The sustainability of the sensor nodes is being controlled using these energy harvesting nodes. The wireless mechanism has been studied at various points during data transfer to the monitoring unit. However, wearable sensors for continuous monitoring are possible for certain technologies, such as RFID, near-field communication (NFC), and resonant antennas. These technologies use low power, provide high data transfer rates, and provide high security. Other than proper data transfer, heat generation and biocompatibility are some attributes prioritized for wearable sensors for healthcare applications.

## 5. Data Analysis for Healthcare

Data processing and analysis involve wearable sensor data collection and processing, which are the early stages of wearable technology computing [163]. The computational task begins after these two steps (i.e., analysis). The data analysis has to infer meaningful information from the collected data. For example, wearable sleep trackers can provide valuable data about company employees. Employees at the insurance company Aetna, for instance, can increase their incentives if their sleep tracker data demonstrate that they get the recommended amount of sleep each night during a predetermined time [164]. In another example, features such as Geolocation, user personalization tendency, and many other data effective and personalized strategies can help devices that heart patients sometimes wear to send data to the concerned person. If an alarming situation occurs, the wearable device will send the signal/information to a doctor and other entities to help the patient as soon as possible. Applications can also provide information to the patient and provide a beneficial tip at the same time when the situation is normal. Practical wearable technology data analysis always leads to increased productivity [164]. For example, wearable devices suitable for employees will provide a variety of data related to productivity. For the most productive hours, the necessary breaks, the health stability, and the specific health needs of employees can be monitored. As a result, companies can help employees achieve their health and productivity goals. Wearable technology data analysis using AI-powered digital health platforms provides community staff with actionable insights into each individual’s activities and behaviour, identifying older adults at high risk of ill health [165]. Most of the wearable sensors and the systems connected are resource-constraint computing devices. 

The implementation of AI techniques in wearable sensor data-handling can be performed with two different computing paradigms [94].

### 5.1. Edge Computing

Edge computing involves data processing and analysis near the data source in health sector applications with wearable sensors and systems. The advantage of this computation is that it can reduce the sensor data latency with high-speed computation and real-time data processing [166]. Moreover, intelligent wearable systems can monitor physiological parameters and infer decisions in real-time. Edge computing can help create a new ecosystem to help improvise communication demands for the following applications:

- Detection of human falls, monitoring stress, and activity recognition;
- Integration of security and privacy mechanisms;
- Health care systems to communicate using 5G/6G technologies;
- For remote treatment and surgery;
- Wearable EMG, ECG, EEG, and many more.

### 5.2. Fog Computing

Smart wearable devices will provide users with comprehensive health data and notifications from sensors to their mobile phones connected in the form of a fog framework [167].

**Figure 11:**
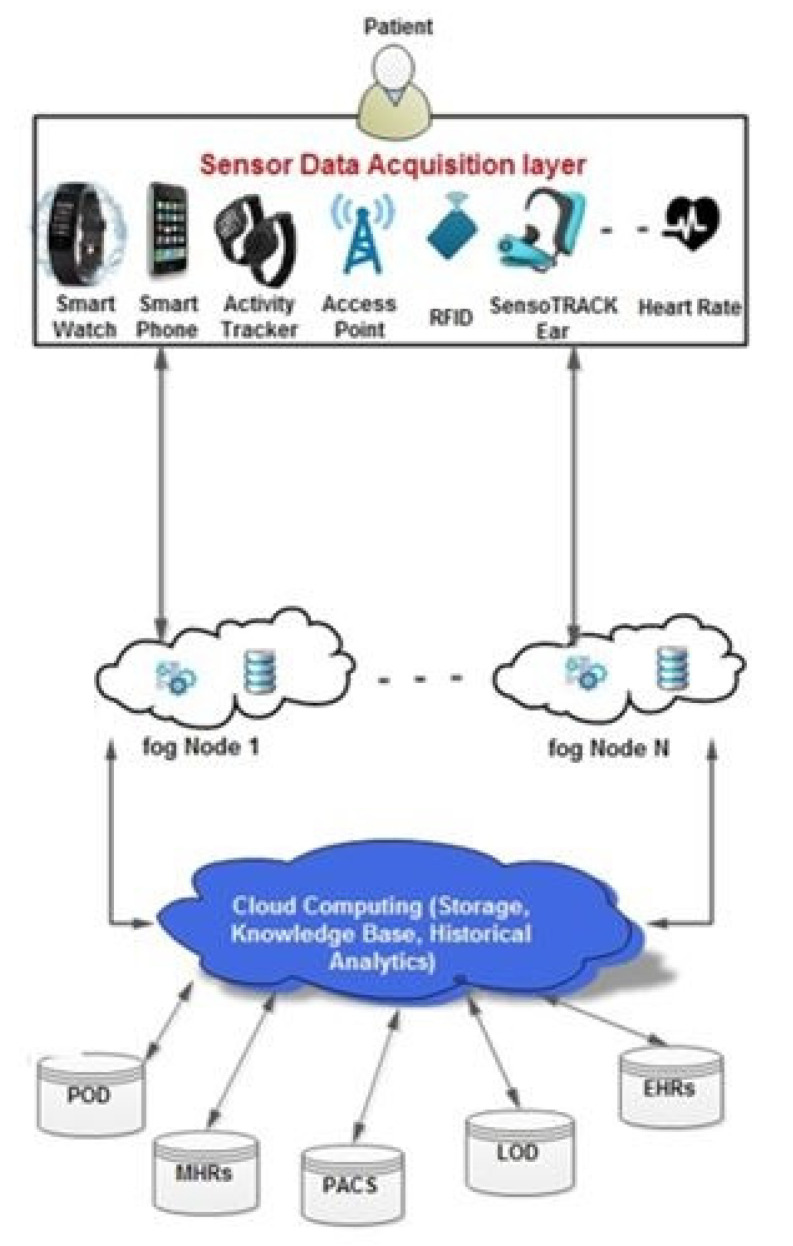
Representation of the components in the fog framework for wearable healthcare systems.

Medical wearable devices generate data and communicate with a medical IoT gateway that mediates between the device and the fog layer. The data collection process uses a particular IoT protocol that connects the wearable device to an IoT gateway. Wearable devices are mainly based on sensor technology, wireless network systems, and protocols such as Wi-Fi, Bluetooth, and GPS. Figure 11 depicts a generic fog framework for data collection and analysis by wearable devices. Exciting developments have resulted from the development of IoT technology in the twenty-first century. Wearables and IoT smart medical devices produce and transmit large amounts of heterogeneous data at high speeds. As a result, the services offered by conventional techniques, such as cloud computing, to manage these data are inadequate. For IoT-based healthcare, edge/fog computing is reportedly expected to generate tremendous interest in the medical area. Real-time analysis and low latency have become increasingly important, contributing to the stringent demand for combining IoT with the edge/fog computing paradigm [168].

## 6. IoT-Enabled System for Healthcare

Wearable technology across many years has allowed the diagnosis of various health characteristics by monitoring using small devices, such as Smart Watches. This technology also has transformed hospital-centric medical systems into patient systems with the help of the IoT [169]. For example, some clinical analyses (e.g., blood pressure measurement, blood glucose level, and O2) can be conducted at home without the help of medical personnel. Clinical data can also be communicated to a medical centre in a remote area using advanced communication services. Emerging technologies, such as machine learning, big data, IoT, wireless communication, mobile computing, and cloud computing, improve health facilities’ availability. The IoT has increased independence and diversified individuals’ ability to interact with the external environment. With the help of various wireless communication protocols and methods, the IoT has become a significant component of global communication. It connects many devices, wireless sensors, home appliances, and electronic devices to the Internet [170].

A simple IoT architecture for wearable technologies includes three components, i.e., a publisher, broker, and subscriber [171]. The publisher represents a community of connected sensors and different scientific gadgets that may match personally or concurrently to record the patient’s crucial statistics. These statistics include the blood pressure, heart rate, temperature, oxygen saturation, ECG, EEG, and EMG. The publisher can send these statistics constantly via communication technology to a broker. The broker is responsible for processing and forwards the obtained records to the cloud. Finally, the subscriber indulges in the non-stop tracking of the patient’s statistics that may be accessed and visualized via a smartphone, computer, or tablet. Herein, the publisher can process those records and deliver comments after the communication of any physiological anomaly or degradation in the patient’s fitness condition. The IoT assimilates discrete additives into a hybrid grid in which a particular cause is devoted to every issue in the IoT system and cloud within the healthcare community. Since the topology for an IoT relies upon the healthcare need and application, it is difficult to indicate a particular structure for the general IoT.

## 7. Cloud Computing for Healthcare

A wide range of applications and services, including patient monitoring and emergency response, can be supported by mobile pervasive healthcare technologies. However, they simultaneously have several difficulties, including data management and storage, resource availability, and interoperability across heterogeneous systems, and problems with unified and universal access. Adopting cloud computing is one potential remedy for all of the issues mentioned earlier. For example, a wearable platform based on open hardware and software can gather motion and heartbeat data and wirelessly store it on an open Cloud infrastructure for observation and further processing. This technology might let older people and others who need regular supervision live independently.

Cloud computing has many applications, but most of them are Internet computing. Connecting to the Cloud is connecting to the Internet, which has become easier with advances in wireless technology. It is easy to see how Cloud computing has driven the rapid spread of wearable technology. Helping people connect to the Internet from any point can allow them to easily access all of their data [172]. Think of the convenience millions of people have gained from using smartphones. We now have access to computers through the clothes we wear. Wearable technology makes it possible to access data in the cloud, generate them, and send them to large data centres. Most impressively, these data can be accessed and collected in real-time.

## 8. Security Issues for Wearable Devices

Internet computing with wearable technology has changed the concept of real-time monitoring. Unfortunately, it has also made healthcare networks more vulnerable to cyberattacks [173]. Internet-based wearable computing can lead to the mishandling of valuable patient information and affect treatment. Some precautions are required when designing a system to prevent these malicious attacks on the IoT system. To avoid attacks, medical and sensor devices in IoT networks must evaluate and use identity authentication, secure boot, failover, rights management, whitelisting, password encryption, and secure pairing protocols [174]. Wearable devices integrated with IoT devices must be combined with secure routing mechanisms and message integrity checks. Because IoT is a connected network in which all users are connected to a server, patient privacy can be compromised if the IoT security service fails. This can be addressed by incorporating advanced security algorithms and encryption to create a more secure environment.

## 9. Design Challenges of the Wearable Healthcare System

Today, we are witnessing the beginning of a medical revolution with wearable devices. Wearable devices with built-in transmission sensors can continuously and non-invasively monitor vital human signs and transfer data to an electronic device for storage, analysis, and visualization [175]. This has been made possible by the fusion of several technologies, including sensing techniques, micro-electromechanical technologies, data analysis, and software development. The design aspects include sensor accuracy, identifying needs related to individuals and business models for market usage, data privacy, and security. The primary topics that have arisen as design challenges in considering sensors as part of wearable devices are privacy issues, moral difficulties, and absence of trust, which are the most well-known among the subjects [176]. In addition, patients’ protection utilizing vision sensors, and remote innovation in homes is challenging since it influences patients’ trust and conduct in IoT gadgets.

People have been pushed to adopt a healthy lifestyle due to the rising frequency of chronic diseases, the ageing population, and the global pandemic. This has sparked the development of cutting-edge personal health equipment and patient-focused software to identify fundamental symptoms, track body functions, support patients during therapy, and administer daily prescriptions. However, due to various sensor integration techniques, various connectivity options, and low power consumption requirements in a portable form factor, the development complexity of these devices has risen as they decrease in size.

## 10. Design Challenges of the Wearable Healthcare System

The design aspects include sensor accuracy, identifying needs related to individuals and business models for market usage, data privacy, and security. The primary topics that arise as design challenges in considering sensors as part of wearable devices are privacy issues, moral difficulties, and absence of trust, which are the most well-known among the subjects. Patients’ protection utilizing vision sensors and remote innovation in homes is a challenge to this plan since it influences patients’ trust and conduct in utilizing IoT gadgets. The deteriorating health of elderly people in general has led researchers to constantly study smart sensing systems. The older view freedom and independence as key, so innovations that advance these perspectives will probably be acknowledged. However, mentalities, such as innovation, nervousness, convictions, and viability, challenge their reception rate. Different difficulties noticed in corporate clients have restricted associations with information, which might guide the improvement of valuable sensors in homes. Answers for utilizing savvy gadgets to upgrade clients’ associations and information usage productivity remain restricted.

## 11. Power Supplies for Wearable Healthcare System: Energy Harvesting Issues

The continuous usage of power for wearable sensors has also been an issue since their utilization in healthcare applications. Although some of the uses require sporadic measurements, ubiquitous measurements require a continuous supply of power. In the case of wireless communication, this power is supplied by batteries, which have limited life. Thus, the energy-harvesting phenomenon is one of the possibilities to deal with this issue, where energy will be generated and supplied simultaneously to operate the sensing system [177,178]. There is a growing need for energy-harvesting technologies to operate autonomous wearable sensors, such as those for the detection of a heartbeat, body temperature, blood pressure, blood sugar level, and body falls [178]. Energy-harvesting devices hold a pivotal position in the field of health monitoring and telemedicine. Researchers are currently focusing on self-powered technologies to devise sustainable energy supplies for portable wearable sensors [179]. In this way, sensing systems can maintain their state in the working environment without the need for an external energy supply. From the sensorial point of view, nanogenerators, such as TENG [180,181] or piezoelectric nanogenerators (PENGs) [182,183], are ideal options that can generate and store an adequate amount of energy for sensing operations. With the variation in the materials and working mechanism of these nanogenerators and nano-harvesters, the output current and voltage also greatly vary. When these energy-harvesting devices are used in electrochemical and strain-sensing applications, their capability is greatly tested in real-time conditions. Due to their enhanced electromechanical characteristics, the use of nanotechnology in developing wearable sensors has recently been growing rapidly [72]. Due to their increased physical strength, their uses as photovoltaics [184], thermo-photovoltaics [185], piezoelectric sensors [186], and ferroelectric sensors [187], thermoelectric sensors [188] and magneto-mechanical sensors [189] can assist in energy harvesting applications. These nanomaterials are capable of offering enhanced ionic transport and electrical conductivity, thus increasing their tolerance for high currents [190]. Other than nanomaterials, different approaches are being used to eliminate the use of batteries in wearable sensing systems. In recent years, FPCBs have been developed and equipped with energy harvesting mechanisms to store energy from renewable sources in supercapacitors and use it for further purposes [91]. Low-cost, flexible antennas are also being considered for wideband low-power operation [92]. Radio-frequency energy tags are also available to harvest energy via an external antenna and the backscattering modulation process [93]. They can help in sending data to the monitoring unit with an adequate data transfer rate and size. The most recent phenomenon is the use of neural networks, where different kinds of functional networks are being employed in addition to the models for comparing their performances [94]. The use of these techniques circumvents the energy-harvesting issue to a great extent while maintaining a high quality of service for the attached sensors and actuators.

## 12. Future Possibilities of Wearable Healthcare Systems

Although a lot of work has been conducted in the field of wearable healthcare sensors in the last decade, there is still some scope to improve the quality of these systems to a great extent. The sensors fabricated for wearable sensing demonstrate the focus on multifunctional applications that operate under discrete working mechanisms. Electrochemical sensing conducted using implantable sensors is still not a common event, and patients have to undergo severe invasive procedures. This increases the risk of unsuccessful operation and minimizes the accuracy of the sensed data [191]. The conjugation of the nanomaterials to build sensors is another area to work on. The conjugation of nanowires can help in integrating their individual properties to obtain enhanced products. The conjugation of the sensors with signal-conditioning circuits should be conducted with flexible printed boards [192,193] that can increase the comfort of the patients. Energy harvesting by wearable sensors should be conducted using multiple arrays to accumulate a large amount of energy. The consideration of an array of sensors can be helpful, especially in the case of the detection of electrochemical ions, such as glucose and proteins. Nanogenerators developed for energy-harvesting purposes should be considered and implemented to generate and harvest energy from the physiological movements of human beings. Regarding the integration of IoT with wearable sensors, artificial intelligence and machine-learning techniques should be used to increase their efficiency in terms of sensitivity, efficient handling of data, scope of improvement, data acquisition, a wider range of applications, and better accuracy. In addition to the use of these nanomaterial-based wearable sensors in the laboratory environment, their commercialization and real-time use are also necessary to determine their performances in terms of sensitivity, robustness, and longevity. Encouragement to academic institutions should be given to open more start-ups to share and sell ideas regarding novel wearable sensing systems. The market for wearable sensors has been estimated to increase to 2.86 billion USD by 2025, with a compound annual growth rate of 38.8% [194]. Some of the sensing prototypes, such as motion sensors, accelerometers, and electrochemical sensors, will be used more to ensure better health. In terms of applications, there are many more areas in which these wearable sensors can be utilized. The attachment of these sensors to the periphery of the human organs and textiles would assist them in being used as pH sensors. Although there is not much change in pH under normal circumstances, an imbalance in the pH level can be caused due to the malfunctioning of the lungs and kidneys. These changes subsequently disrupt the acid–base balance, which leads to issues such as acidosis and alkalosis. Researchers have worked to develop both body-attached and implantable flexible sensors to measure the pH level over a wide range. Some of the advantages of wearable pH sensors are their excellent flexibility, good integration capability, high sensitivity, quick response time, and low recovery time [195]. Implantable pH meters have the edge over externally attached prototypes in this case, as they can measure the local changes in pH due to the implantation of other wearable sensors. While dealing with elderly people, pH sensors would be advantageous if clinically demonstrated, as pH modifications can result from potentially serious diseases. A wide range of printing techniques have been used in the fabrication process to form thin-film pH sensors [196,197]. The working mechanism of these pH sensors is based on the changes in impedimetric [198,199], potentiometric [200,201], and microfluidic [202,203] technologies to track the pH levels of the skin. The state-of-the-art technology that the researchers are focusing on is the minimization of the influence of volatile organic compounds on the response of pH sensors. The sensors are coated with selective materials that are resistant to the endogenous and exogenous compounds produced in the human body. The use of textile-based wearable radio-frequency identification (RFID) tags for biomedical applications is another area that should be further researched. These types of wearable sensing technology are in high demand due to their tremendous prospects in e-health, IoT, and smart cities [204]. While these prototypes can be easily operated using low-cost antennas, they can help in performing maintenance-free communication links with the human body. The real-time monitoring of the physiological signals using these devices is very significant, as they are deployed as cost-effective substitutes for chipped RFID sensors. Some of the advantages of using this technology are the avoidance of electronic chips and an increase in the number of bits for data transmission purposes. These sensors have also been built with printing techniques, such as screen printing with sensing materials, such as CNTs, PEDOT, and metallic oxides [205]. In order to use them for wearable applications, such as anti-counterfeiting systems and laundry labels, researchers have been studying some of the essential parameters of these devices, such as frequency [206,207]. A wide range of conductive fabrics have been used to form single and multi-layered structures that operate on the resonance of the frequency values.

Other areas where an extension of the use of the wearable sensors can be conducted include stress [208,209] and dermally implanted [210,211] sensors to determine the emotional condition and perception of a person with respect to their responses towards environmental stimuli. These sensors contribute equally to the well-being of a person, as they can detect the issues faced by a person in a particular environment. With competition growing exponentially in every sector, the physical and mental well-being of human beings is of utmost importance. These stress sensors could be used in real-time applications to potentially help determine and classify the job sectors with high stress. Eventually, the manpower in those job areas could be increased to reduce the stress on a particular person. This would not only help to improve the quality of life, but allow people and investing sectors to choose their career paths. The inclination to use wearable sensors can be increased by standardizing the protocols in using these sensors as per the environment, age, gender, and preceding health conditions. Some of the other aspects in which these wearable sensors can be deployed are for communication [212,213] and energy-harvesting [214,215] applications. This will not only increase the functionality of the sensors, but improve the quality of the sensing systems in terms of lower cost, higher robustness, and lower complexity. The inclusion of these applications would involve a change in the structure of the current wearable sensors. The prototypes need to be array-based, where each section should be assigned for a target application. A thorough survey should be carried out on the combination of the materials to determine which nanomaterials and polymers are suited to allow the maximum number of uses. The use of flexible printed circuit boards is another area that should be researched to form arrays for the inclusion of these applications [216,217]. The use of commercial products, such as smartwatches, smart glasses, and smart fabrics, for people to stay fit and maintain a quality of life is increasing. The end-to-end portfolio of the sensors will be fabricated using MEMS and printing techniques [218]. These sensors are being used in various sectors, such as the consumer, defence, healthcare and industries. The presence of the COVID-19 pandemic has further increased the usage among human beings due to the lack of physical movement outside their homes.

## 13. Conclusions

This paper provides a substantial review of the wearable sensors that are used for healthcare applications. The design, development, and utilization of wearable sensors have been provided in the preceding sections. These sensors are capable of providing information on all the organs of the human body in a quick and efficient manner. Both the types of sensors developed in the laboratory and industries have been explained in this paper. The integration of the sensing prototypes with wireless communication protocols to form the entire wearable sensing system has been highlighted. Then, the research related to the sensed and processed information has been elucidated in terms of data analytics, IoT-enabled systems, cloud computing, and security issues. Finally, some of the challenges related to the current wearable systems and energy-harvesting issues have been mentioned.

## Figures and Tables

**Table 1:** Comparison of some commonly used wireless protocols for wearable healthcare applications [162].

Type	ZigBee	Bluetooth	Wi-Fi	Wi-Max
Range	100 m	10 m	5 km	15 km
Data rate	50–500 kbps	1–3 Mbps	1–450 Mbps	75 Mbps
Band width	2.4 GHz	2.4 GHz	2.4, 3.7 and 5 GHz	2.3, 2.5 and 3.5 GHz
Network topologies	Star, Mesh, Cluster trees	Star	Star, Tree, P2P	Star, Tree, P2P
App	Wireless sensors	Wireless sensors	PC-based data acquisition	Mobile internet
